# Enhancing water stress tolerance improves fitness in biological control strains of *Lactobacillus plantarum* in plant environments

**DOI:** 10.1371/journal.pone.0190931

**Published:** 2018-01-05

**Authors:** Núria Daranas, Esther Badosa, Jesús Francés, Emilio Montesinos, Anna Bonaterra

**Affiliations:** Institute of Food and Agricultural Technology-CIDSAV-XaRTA, University of Girona, Girona, Spain; Karnatak University, INDIA

## Abstract

*Lactobacillus plantarum* strains PM411 and TC92 can efficiently control bacterial plant diseases, but their fitness on the plant surface is limited under unfavourable low relative humidity (RH) conditions. To increase tolerance of these strains to water stress, an adaptive strategy was used consisting of hyperosmotic and acidic conditions during growth. Adapted cells had higher survival rates under desiccation than non-adapted cells. Transcript levels and patterns of general stress-related genes increased immediately after the combined-stress adaptation treatment, and remained unaltered or repressed during the desiccation challenge. However, there were differences between strains in the transcription patterns that were in agreement with a better performance of adapted cells of PM411 than TC92 in plant surfaces under low RH environmental conditions. The combined-stress adaptation treatment increased the survival of PM411 cells consistently in different plant hosts in the greenhouse and under field conditions. Stress-adapted cells of PM411 had similar biocontrol potential against bacterial plant pathogens than non-adapted cells, but with less variability within experiments.

## Introduction

The development of practical strategies for the sustainable management of plant diseases to minimize the use of environmentally aggressive pesticides pose a challenge to worldwide crop production [1–2]. The number of crops without efficient protection methods has increased in recent years and this fact has stimulated an increasing demand of beneficial plant-microbe interactions to withdraw biotic and abiotic stresses [3–5]. Thus, there is a need for finding new and more effective biological control agents (BCA) and biostimulants, and also for optimizing the methods by which these new products are made viable, durable, robust and economical [6–8]. *Lactobacillus plantarum* strains have been reported as novel BCA for bacterial diseases control such as fire blight of rosaceous plants [9–10].

The establishment of the BCA on plant organs, prior to the arrival of the pathogen, is a key factor to achieve an efficient biocontrol of aerial plant diseases. In many BCA, a population decline is often observed after application to plants, thus reducing biocontrol efficiency [11–12]. This decline of population is due to the shift from laboratory culture conditions to the growth-limiting plant surface [13]. In addition, microorganisms are exposed on the phyllosphere to ultraviolet radiation, nutrient limitation, and fluctuating water availability [14–15]. These reasons may explain the limited efficacy of lactic acid bacteria like *L*. *plantarum* strains observed under non-optimal environmental conditions.

However, lactic acid bacteria (LAB) are able to tolerate several stresses (pH, salt, heat, inhibitory compounds) [16], and as in other bacteria, *L*. *plantarum* have several mechanisms to enhance survival under stressful environments [17–19]. These mechanisms include the synthesis of chaperone proteins (GroES/GroEL and DnaK/DnaJ/GrpE), which assist the folding of misfolded proteins, and proteases (Clp family of proteins, such as ClpA, ClpB, ClpC, ClpE, ClpL and ClpX), which degrade damaged proteins; both regulated mainly by the transcriptional repressors CtsR and FtsH [20–21]. Also, small heat shock proteins (sHSP) and cold shock proteins (CSP) function as folding chaperones, and play an important role in maintaining membrane integrity under stress conditions. Cells exhibiting an increased synthesis of these proteins have shown a greater ability to survive under stress conditions [22–24]. In addition to stress related proteins, bacteriocins (e.g. plantaricins) may confer competitive advantages to *L*. *plantarum* [25–26] and their synthesis is affected by growth conditions and stress [27]. Also, the adhesion-like factor EF-Tu may function as an “envelope associated protein”, which can be released from the cell when they experience osmotic shock [28–29]. As it has been suggested, transcriptional profiling of stress-related, bacteriocin-encoding and adhesion factor genes could be used as a tool to evaluate the suitability of adaptation strategies, and to compare strains adaptability to a stressful environment [30–33].

Different strategies have been used to increase BCA adaptation to stress and to improve its epiphytic fitness upon delivery to the field. For example, cells have been adapted during its cultivation to a sub-lethal dose of a specific physical or chemical stress [34–36] or to heat shock [37–38]. These adaptation treatments can significantly improve the subsequent performance of the microorganism under suboptimal conditions, not only to the specific stressing factor used during adaptation, but also to a range of different stresses, a phenomenon known as cross-protection [39–40]. In lactic acid bacteria it has been described that the exposure to a single stressor is commonly associated to the cross-tolerance to other stressors like acid [41], heat shock [42] or desiccation [43]. The development of cross-tolerance to stress has been described in *L*. *plantarum* strains from food processing and probiotics [24, 44]. However, there are no reports on tolerance to stress in *L*. *plantarum* biological control strains of plant diseases.

In the present work, we wanted to physiologically improve the fitness of *L*. *plantarum* strains PM411 and TC92, by means of a strategy of tolerance to desiccation stress and low relative humidity conditions that are common problems onto plant surfaces. We used a procedure consisting of the adaptation of cells to salt and/or acidic pH conditions during inoculum preparation. The response of both strains to stress was compared by means of: (1) the effect of different adaptation treatments on cell inactivation during desiccation stress, and on plant surfaces under controlled environment conditions at low relative humidity (RH), and (2) the transcriptional pattern of selected genes after the adaptation treatment and during the desiccation challenge. Finally, (3) the effect of the adaptation treatment on the survival of PM411 on different plant surfaces under greenhouse and field conditions and on the efficacy of biocontrol was studied.

## Materials and methods

### Bacterial strains and growth conditions

*L*. *plantarum* strains PM411 and TC92 were isolated from pear and tomato, respectively, as described previously [10, 45]. Strains were cultured in Man, Rogosa and Sharpe broth (MRS) (Oxoid; Unipath Ltd., Basingstoke, Hampshire, England) at 30°C in an orbital shaker at 80 rpm. Stock cultures were stored at -80°C in MRS containing 20% (v/v) glycerol. To monitor culturable populations of PM411 and TC92 in plant colonization studies, spontaneous mutants resistant to rifampicin (PM411R and TC92R) were used [10]. *Erwinia amylovora* EPS101 isolated from an infected shoot of a Conference pear tree in Lleida (Spain) [35] and *Xanthomonas fragariae* CECT549 (Colección Española de Cultivos Tipo) were used in pathogen inoculation experiments. Bacterial suspensions of the pathogens were obtained from ultrafreeze-preserved cultures (-80°C) grown overnight at 25°C in Luria–Bertani (LB) agar for *E*. *amylovora* and in medium B agar [46] for *X*. *fragariae*. Colonies were scraped from the agar surface and suspended in sterile distilled water. The culture was adjusted to a cell density corresponding to 1 x 10^8^ CFU mL^-1^ (OD_600_ of 0.12 and 0.40 for *E*. *amylovora* and *X*. *fragariae*, respectively) and diluted with sterile distilled water to obtain an appropriate concentration.

### Adaptation treatments

**Fig 1:**
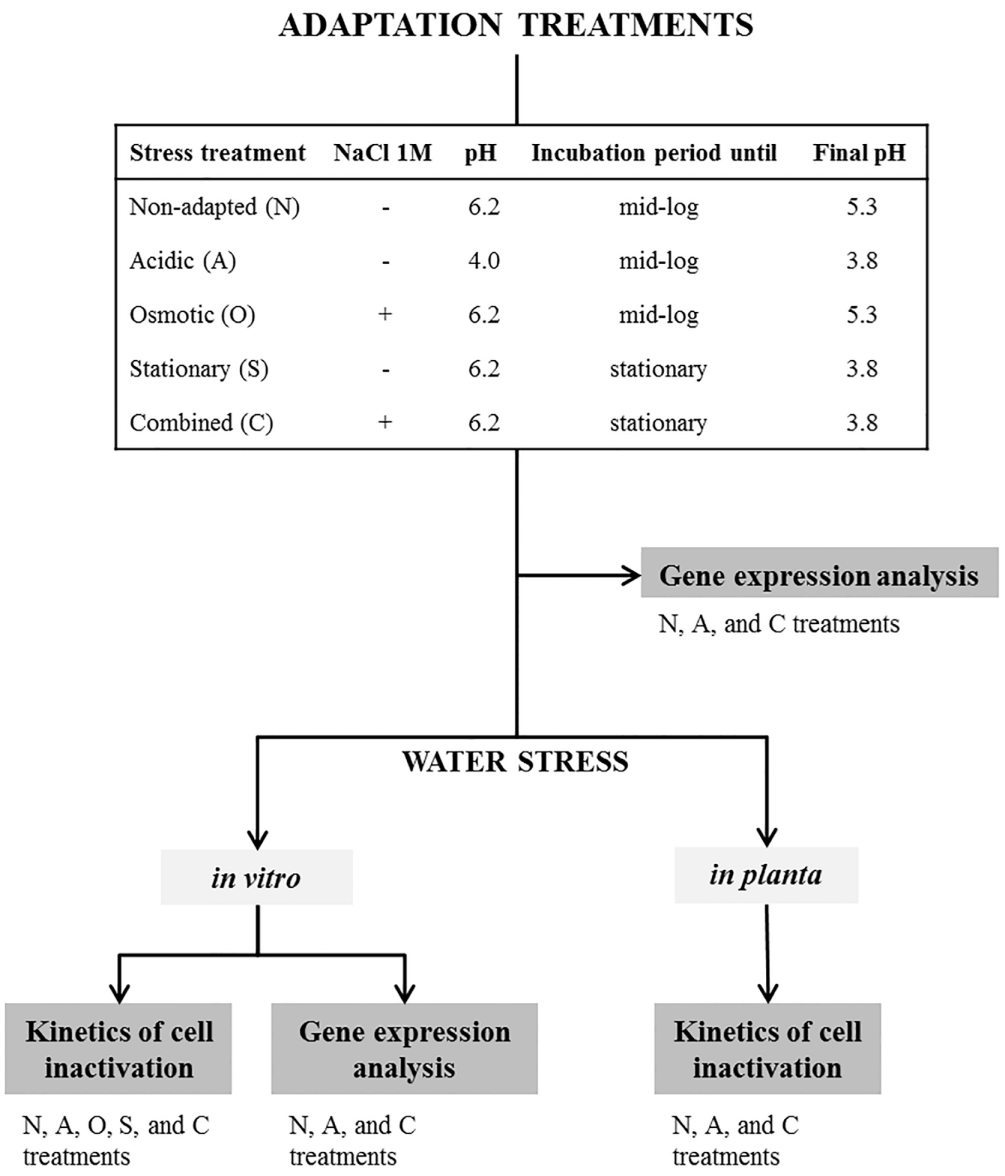
Schematic representation of the adaptation treatments and water stress challenge, and the analysis performed in *L*. *plantarum* PM411 and TC92 strains. Gene expression of cells was studied in non-adapted (N), acidic (A), and combined-stress (C) conditions after the adaptation treatments. After the treatments (N, A, and C) cells were exposed to water stress (i) *in vitro* for the study of cell inactivation kinetics and gene expression, and (ii) *in planta* for the study of cell inactivation kinetics.

PM411 and TC92 cells were cultured overnight in MRS broth until an OD_600_ of 0.8–1.0, harvested by centrifugation (10000 x g, 10 min at 10°C), and washed in 50 mM sterile phosphate buffer (PBS, pH 7.0). Washed cells were resuspended in the corresponding adaptation medium at an initial OD_600_ of 0.2, or cultured accordingly. The adaptation treatments performed were acidic, hyperosmotic, stationary phase, combined-stress, and non-adapted (Fig 1). For the acidic treatment (A), cells were cultured until mid-log phase (final conditions OD_600_ = 0.6, pH 3.8 ± 0.2) in MRS broth at pH 4.0 (modified by the addition of HCl 1N). For the hyperosmotic treatment (O), cells were cultured until mid-log phase in MRS broth (pH 6.2) amended with NaCl 1 M (final conditions OD_600_ = 0.6, pH 5.3 ± 0.2). For the stationary phase treatment (S), cells were cultured in MRS broth at pH 6.2 until stationary phase (final conditions OD_600_ = 1.0, pH 3.8 ± 0.2) (no pH control). For the combined-stress treatment (C), cells were cultured in MRS broth (pH 6.2) amended with NaCl 1 M until stationary phase (final conditions OD_600_ = 1.0, pH of 3.8 ± 0.2). For non-adapted treatment (N), cells were cultured in MRS broth (pH 6.2), until mid-log phase (final conditions OD_600_ = 0.6, pH = 5.3 ± 0.2). All treatments were incubated at 30°C with shaking at 80 rpm. Three independent biological replicates were prepared for each *L*. *plantarum* strain (PM411 and TC92) and treatment combination. Two independent experiments were performed.

### Effect of adaptation treatments on cell survival during desiccation

Previously to the water stress challenge, cells were obtained from cultures grown under different adaptation conditions, as described above. In the first experiment, the adaptation treatments performed were A, O, S, C, and N, and subsequently cells were exposed to desiccation along a period of 6 days (long-term). In the second experiment, the adaptation treatments were A, C, and N, and the exposure to desiccation was along 5 h (short-term). In both experiments, cells from culture aliquots (25 ml) were harvested by centrifugation (10000 x g, 10 min at 10°C), washed once in PBS and resuspended in 25 ml of sterile PBS (pH 7.0). In the case of O and C cells, the sterile PBS used to wash cells was amended with NaCl to a final concentration of 1 M. Then, aliquots of 1 ml were distributed into 1.5 ml Eppendorf tubes and harvested by centrifugation at 6000 x g for 5 min. The supernatant was discarded and the pellet containing cells was dried in a vacuum desiccator using silica gel as desiccant as described [34]. The tubes were maintained opened in the desiccator at 25°C. In the first experiment, samples for cell survival analysis (an Eppendorf tube) were taken at 1, 2 and 6 days after the start of the desiccation challenge. In the second experiment, tubes were taken at 0.5, 1, 1.5, 2, 3, 4 and 5 h. The experimental design consisted of three independent biological replicates of each adaptation treatment and date of assessment, for each strain and experiment. For cell survival analysis, the pellets were resuspended in 1 ml of sterile PBS (pH 7.0) and appropriate dilutions of the suspensions were seeded onto MRS agar plates (Panreac, Barcelona, Spain) using a spiral plater system (Eddy Jet; IUL Instruments, Barcelona, Spain). Plates were incubated at 23°C for 48 h. Colonies were counted using an automatic counter system (Countermat Flash; IUL Instruments) and counts were transformed to CFU ml^-1^ of culture. Non-desiccated cells of each adaptation treatment (N, A, O, S, and C) served as controls. The inactivation of cells for each treatment was calculated as log N_0_/N, where N_0_ is the initial number of unstressed cells (control) and N the number of survivors.

### Effect of adaptation treatments on survival of *L*. *plantarum* under low relative humidity conditions

#### Apple flowers

Open blossoms were collected from an experimental field of Golden Smoothie apple cultivar near Girona (Mas Badia Agricultural Experiment Station), taken to the laboratory under refrigeration, and used within 24 h. The procedure was essentially as described [47]. Briefly, detached flowers were maintained with the cut peduncle submerged in a sucrose solution in Eppendorf tubes and placed in transparent plastic boxes. Flowers were treated with a hand sprayer to the runoff point (0.4 ml per flower), with suspensions of PM411R and TC92R cells (10^8^ CFU ml^-1^) from A, C, or N adaptation treatments. Adapted and non-adapted cells were obtained as described above, and resuspended in quarter-strength Ringer’s solution by setting the OD_600_ of cell suspensions to 0.25 (confirmed by colony counts, 10^8^ CFU ml^-1^). After the corresponding treatment, inoculated flowers were placed at 25±1°C, with 16 h of fluorescent light/8 h dark and under low relative humidity (RH) conditions (50%). RH conditions were obtained in controlled environment chambers (SGC097.PFX.F, Fitotron, Sanyo Gallenkamp plc, UK) by placing CaCl_2_ into the chamber as a humidity absorber. The experimental design consisted of three independent biological replicates of each adaptation treatment and date of assessment for each strain. Two independent experiments were performed. Samples of 5 flowers from each replicate were taken for monitoring population levels of PM411R or TC92R at 1, 2 and 6 days post inoculation. Flowers were homogenized with 20 ml of sterile PBS (pH 7.0) and 0.1% peptone using a stomacher (Masticator; IUL Instruments). Sample homogenates were diluted and appropriate dilutions were seeded onto MRS agar plates supplemented with 50 μg ml^-1^ of rifampicin (Sigma, Missouri, USA) to counter-select the corresponding strain inoculated. Plates contained 10 μg ml^-1^ of econazole nitrate salt (Sigma, Missouri, USA) to avoid fungal growth. Plates were incubated at 23°C for 48 h and colonies were counted. The population levels of PM411R or TC92R were expressed as CFU per flower. A sample of recently inoculated flowers by each adapted (A and C) and non-adapted (N) strain served as a control. The inactivation index for each treatment was calculated as described above.

#### *Prunus* leaves

*Prunus amygdalus* × *P*. *persica* plants of the rootstock GF-677 were obtained by micropropagation (Agromillora, Barcelona, Spain). Plants of 20 cm in length were grown in 10-cm-diameter plastic pots having 6 to 10 young leaves, and were maintained in a greenhouse at 26 ± 2°C, 60 ± 10% of RH and a 16-h photoperiod, and were fertilized once a week with a 200 ppm N/P/K solution (20:10:20) and standard insecticide and miticide sprays were applied until use. Plants were inoculated with 10^8^ CFU ml^-1^ cell suspensions of A, C, or N adapted PM411R and TC92R cells using a microsprayer until runoff (10 ml per plant). Cell suspensions were prepared as described above. Inoculated plants were placed into transparent plastic boxes at 25°C, with 16 h of fluorescent light / 8 h dark and under low (60–70%)-RH conditions. Low RH conditions were obtained by placing CaCl_2_ into the boxes as a humidity absorber. The experimental design consisted of three independent biological replicates (5 plants per replicate) of each adaptation treatment and date of assessment for each strain. Two independent experiments were performed. For monitoring population levels of PM411R or TC92R five leaves were taken from each replicate at 1, 2, 3, 4 and 7 days post-inoculation. Samples were processed and calculations were done as described above.

### Transcriptional analysis during adaptation conditions and desiccation challenge

Expression patterns of PM411 and TC92 cells obtained immediately after growth under different adaptation treatments (A, C, and N) and after desiccation challenge were analysed. Total RNA was extracted from PM411 and TC92 cells grown under three adaptation conditions (A, C, and N), and exposed or non-exposed to desiccation challenge. A, C, and N cells were obtained following the procedure described above, immediately after growth (non-desiccated) or at different times during desiccation (0.5, 1, 1.5, 2, 3, 4 and 5 h) (Fig 1). At every sampling time dehydrated cells were suspended in 1 ml of sterile PBS (pH 7.0). RNA from 500 μl of cell suspensions was immediately stabilized in two volumes of RNA Protect Bacteria Reagent (Qiagen, Hilden, Germany). Total RNA was isolated according to the procedure recommended by Qiagen with minor modifications, involving enzymatic lysis together with proteinase K digestion, followed by mechanical disruption. The pellet was resuspended with 200 μl TE buffer containing 15 mg ml^-1^ lysozyme (Sigma). Besides, 20 μl of proteinase K and 6 μl of mutanolysin (Sigma) were added and samples were incubated at 37°C for 45 min with shaking at 300 rpm. The mechanical disruption was performed adding 50 mg of acid-washed glass beads (Sigma, 150–600 μm) to the sample using the Tissulyser II instrument (Qiagen) at frequency of 30 s^-1^ for 5 min. Extracted total RNA was purified with the RNeasy Mini Kit (Qiagen) according to the manufacturer’s instructions and RNA was resuspended in 50 μl RNase free water. The concentration and purity of RNA was assessed by spectrophotometric measurements using NanoDrop ND-1000 Spectrophotometer (Thermo Fisher Scientific, Waltham, USA). Residual DNA was removed using the Turbo DNA-free kit (Applied Biosystems, Foster City, USA) and cDNA was synthetized from RNA using the High Capacity cDNA Reverse Transcription Kit (Applied Biosystems) according to the manufacturer’s instructions. The absence of chromosomal DNA contamination was confirmed by minus-reverse transcriptase control in quantitative real-time PCR (qPCR).

Quantitative real-time PCR was carried out in a 7500 Real-Time PCR System (Applied Biosystems) to assess the transcriptional level of *L*. *plantarum* genes associated to: (i) stress response, i.e. encoding molecular chaperones (*dnaK* and *groEL*), Clp proteins (*clpC* and *clpB*), small heat shock proteins (*hsp1* and *hsp3*), cold shock proteins (*cspP* and *cspL*), stress factors (*ctsR* and *ftsH*), and small redox protein (*trxB1*); (ii) adhesion factor protein (*efTU*), and (iii) plantaricin synthesis (*plnE* and *plnJ*). Optimized reactions included 10 μl SYBR^®^ Green PCR Master Mix (Applied Biosystems), 7 μl RNase-free water, 1 μl of each forward and reverse primer (Table 1) at 2 μM, and 1 μl cDNA in a final volume of 20 μl. The thermal cycling conditions were as follows: 10 min at 95°C for initial denaturation; 50 cycles of 15 s at 95°C, and 1 min at 60°C; and a final melting curve program of 60 to 95°C with a heating rate of 0.5°C/s. Each qPCR assay included duplicates of each cDNA sample, no-template and RNA controls to check for contamination. Measures were taken from each condition from three independent biological cultures. The lactate dehydrogenase D gene (*ldhD*) was used as internal control for data normalization [53].

**Table 1:** Function of genes and primer sequences used in the transcriptional response study.

Function	Gene	Forward primer (5’—3')	Reverse primer (5’—3')	Reference
Chaperone proteins	*dnaK*	TCAACCGTGTCACCCAAGTA	TCCTTCAGTTGTGGCATTCA	[31]
	*groEL*	ACCGGATTGAAGATGCTTTG	AACCAGCATTTTCAGCGATT	[31]
Proteases	*clpB*	AGTTACCGGCGTCCATACTG	GACTCAAAGCCGTCTCAAG	[48]
	*clpC*	ATCCTTTCCTCGCGAATTTT	TGGCGTTCCTTCAGTCTTCT	[49]
Small heat shock proteins	*hsp1*	AGGTTGATGTCCCTGGTATTG	TTAAGACACCGTCAGCTTGG	[48]
	*hsp2*	TTACCTTCGCTATCCCGCAAC	CGGTGAAGTATGCTGACGAA	[50]
	*hsp3*	ATCCGCAGCTGCCTTCTTT	CGCGAGTGAACGTCAAACTG	[31]
Cold shock proteins	*cspP*	TACTGGTGAAGATGGCAACG	GAACAACGTTAGCAGCTTGTGG	[44]
	*cspL*	GTGAAGACGGTACCGATGTCTT	GTGGTTGAACGTTCGTTGCT	[44]
Stress factors	*ftsH*	GCAGCTACCTTCGAAGAATCCA	GGGAAACTTGGTTCAGCAACA	[51]
	*ctsR*	AATTTGGTCGATGATGCTGATG	TAAGTCCCGGTCCGTTAATCC	[51]
Small redox protein	*trxBI*	ATGGCAAAGAGTTACGACG	CCCACCATAGATTCCGCGAT	F: [52] R: this study
Adhesion protein	*efTU*	CCACGTAATAACGCACCAAC	TTCTGGTCGTATCGATCGTG	[29]
Plantaricin synthesis	*plnE*	GTTTTAATCGGGGCGGTTAT	ATACCACGAATGCCTGCAAC	[32]
	*plnJ*	TAAGTTGAACGGGGTTGTTG	TAACGACGGATTGCTCTGC	[32]
D-lactate dehydrogenase	*ldhD*	ACGCCCAAGCTGATGTTATATC	AGTGTCCCACGAGCAAAGTT	[53]

Expression patterns of PM411 and TC92 cells obtained immediately after growth under different adaptation treatments (A, C, and N) were analysed and the normalized expression values of selected genes were quantified and log_2_ transformed. Standard curves for quantification of gene expression were created using decimal dilutions of recombinant plasmid DNA (target sequences were cloned into a vector pSpark^®^ in *Escherichia coli* DH5α cells) corresponding to copy numbers ranging between 10^2^ and 10^6^. Ct values in each dilution were measured in triplicate using the optimized qPCR as described above and a negative non-template control was included in each run. Ct values were plotted against the logarithm of their initial template copy numbers and each standard curve was generated by a linear regression of the plotted points.

Besides, the effect of desiccation stress (1, 1.5, 2, 3 and 4h under desiccation) on the transcriptional response of selected genes of PM411 and TC92, depending on the adaptation treatments (A, C, and N), was analysed. In this case, the comparative critical threshold (ΔΔCt) method was used to assess the relative transcriptional level. Similar amplification efficiencies of all genes primer pairs were checked making the ΔΔCt method appropriate to calculate the relative expression (RE) level. The ΔCt of non-desiccated cells, at the corresponding cell-adaptation method (A, C, and N), was used as the calibrating condition to calculate RE level. Genes were considered to be up- or down-regulated if their RE levels were at least twofold (RE level = 2^1^ or 2^−1^) higher or less than the calibrator condition [54, 55].

### Effect of combined stress adaptation treatment on the dynamics of population of PM411 on plant surfaces

Two experiments under greenhouse conditions and two field trials at the Mas Badia Agricultural Experiment Station (Girona, Spain) were performed.

#### Greenhouse experiments

Experiments were conducted in potted plants (10-cm-diameter plastic pots) of cv. Darselect strawberry and cv. Hayward kiwifruit. Plants were used when had 30 to 40 cm in length and 10 to 15 young leaves in the case of kiwifruit plants and 5 to 8 leaves per crown in strawberry plants. Plants were spray inoculated to runoff (6 and 20 mL per strawberry and kiwifruit plants, respectively) with a suspension of PM411R. The inoculum of the biological control agent was prepared as previously described, either adapted with the combined treatment (C) or non-adapted (N). Cells were harvested by centrifugation and resuspended in quarter-strength Ringer’s solution to 5 x 10^7^ CFU mL^-1^. After the corresponding treatment, plants were maintained in a greenhouse at 26 ± 2°C, 70 ± 10% of RH and a 16-h photoperiod. Three replicates of three plants per replicate were used for each adaptation treatment. Sample collection for monitoring population levels of PM411 was performed at 0, 1, 2, 3 and 6 days. Samples of three leaflets for strawberry plants and three leaves for kiwifruit plant were taken from each replicate of the corresponding treatment and sampling date.

#### Field experiments

Experiments were conducted in plots of cv. Golden Smoothie apple and cv. Comice pear trees. Treatments were distributed in a completely randomized block design with three replications of each treatment and 7 trees per replicate. In each tree two branches containing blossoms were tagged. Treatments corresponded to suspensions of PM411 non-adapted (N) or adapted (C). In both treatments cells were harvested by centrifugation and diluted in quarter-strength Ringer’s solution to 5 x 10^7^ CFU mL^-1^. Two strategies were assayed, one doing a single application of bacteria to trees on 29 March 2017 (cv. Comice pear) and on 5 April 2017 (cv. Golden Smoothie apple), and in the second strategy two applications were performed (29 March and 4 April 2017 in the case of pear trees, and 5 April and 11 April 2017 in the case of apple trees). Open blossoms from tagged branches were spray inoculated until near runoff with the bacterial suspension using a hand-held 5 mL sprayer (3 mL per blossom). Weather conditions in the field plots were measured with a weather station located in the experimental field (Mas Badia, La Tallada d’Empordà) (Temperature, RH and rainfall). Sample collection for monitoring PM411 levels was performed at 0, 1, 2, 5, 6 and 7 days. Samples of two blossoms (4–6 flowers and accompanying leaves) were taken from each replicate of the corresponding treatment and sampling date. All the samples (leaves from the greenhouse experiment and blossoms from the field experiment) were homogenized in a sterile plastic bag with 30 mL of 0.05 M phosphate buffer (pH 7.0) and 0.1% peptone under shaking in an orbital shaker at 150 r.p.m. for 30 min at 4°C. The extract was concentrated 10-fold by centrifugation at 10000 g for 10 min. The extract was serially diluted and appropriate dilutions were seeded onto MRS agar plates supplemented with 50 μg ml^-1^ of rifampicin and 10 μg ml-1 of econazole nitrate salt and population levels of PM411 were assessed as described.

### Effect of combined stress adaptation treatment on efficacy of disease control

Two different assays were performed to control angular leaf spot of strawberry (*X*. *fragariae*) and fire blight of apple and pear (*E*. *amylovora*).

#### Angular leaf spot disease of strawberry

Plants were sprayed to runoff with 10 ml of a suspension of adapted or non-adapted cells of PM411. After 24 h, plants were inoculated with a suspension of Xf at 1x10^8^ CFU ml^-1^. Pathogen suspension was prepared in distilled water with diatomaceous earth (1 mg ml^-1^) and applied using a hand-sprayer (at a pressure of 20 psi) to runoff with 6 ml of the suspension. After inoculation, plants were maintained for 48 h in plastic bags to allow a high relative humidity conditions. Then, plant material was maintained in the quarantine greenhouse at 26°C (± 2°C), 60% ± 10 of RH and a 16 h light-8 h dark photoperiod. The experimental design consisted of three replicates of two plants per replicate for each treatment. Kasugamycin treated (Kasumin, Lainco, Barcelona, Spain) (80 mg l^-1^) and non-treated plants were included as controls.

Two experiments were performed. After two weeks from the pathogen inoculation the severity of infections was determined per each replicate. Severity of infections was determined as the level of infection of each leaf, according to the following scale: 0, no symptoms; 1, presence of small spots of necrosis (<25% of the leaf surface); 2, necrosis progression through the leaf (25–50%) and; 3, leaf mainly affected by necrosis (>50%). The mean severity level per leaf was calculated per each replicate of two plants.

#### Fire blight control

Open blossoms of pear and apple treated in the experimental orchard plot (see above) were collected at 1 day after treatment, to perform efficacy assays under controlled environmental conditions. The individual flowers used for inoculation with *E*. *amylovora* were prepared as described [35], in single Eppendorf vials [47]. The hypanthium of flowers was then inoculated with 10 μL of a suspension of *E*. *amylovora* at 10^7^ CFUmL^-^1. The inoculated flowers were again placed in plastic boxes, and incubated at 25°C and high RH for 5 days. The experimental design consisted of three replicates of five flowers per replicate for each treatment. Kasugamycin treated (80 mg l^-1^), and non-treated controls inoculated with the pathogen alone were included. Two experiments were performed. Severity of infections on flowers was evaluated per each replicate after 5 days of pathogen inoculation. The severity of infections was determined as the level of infection of each inoculated flower according to a scale from 0 to 3 in function of the symptoms observed: 0, no symptoms; 1, partial hypanthium necrosis; 2, total hypanthium necrosis; 3, necrosis progression through peduncle.

### Data analysis

To test the significance of the effect of adaptation treatments on inactivation of PM411 and TC92 to desiccation *in vitro*, and on flowers and leaves, and to test the effect of adaptation treatment of PM411 on the population level on plant surfaces and on biocontrol efficacy against *X*. *fragariae* and *E*. *amylovora*, a one-way analysis of variance (ANOVA) was performed. Means of the index of inactivation were separated according to the Tukey's test at P ≤ 0.05. The statistical analyses were performed using GLM procedure of the PC-SAS (version 9.1; SAS Institute Inc., Cary, NC).

## Results

### Effect of adaptation treatments on survival of cells during desiccation

**Fig 2:**
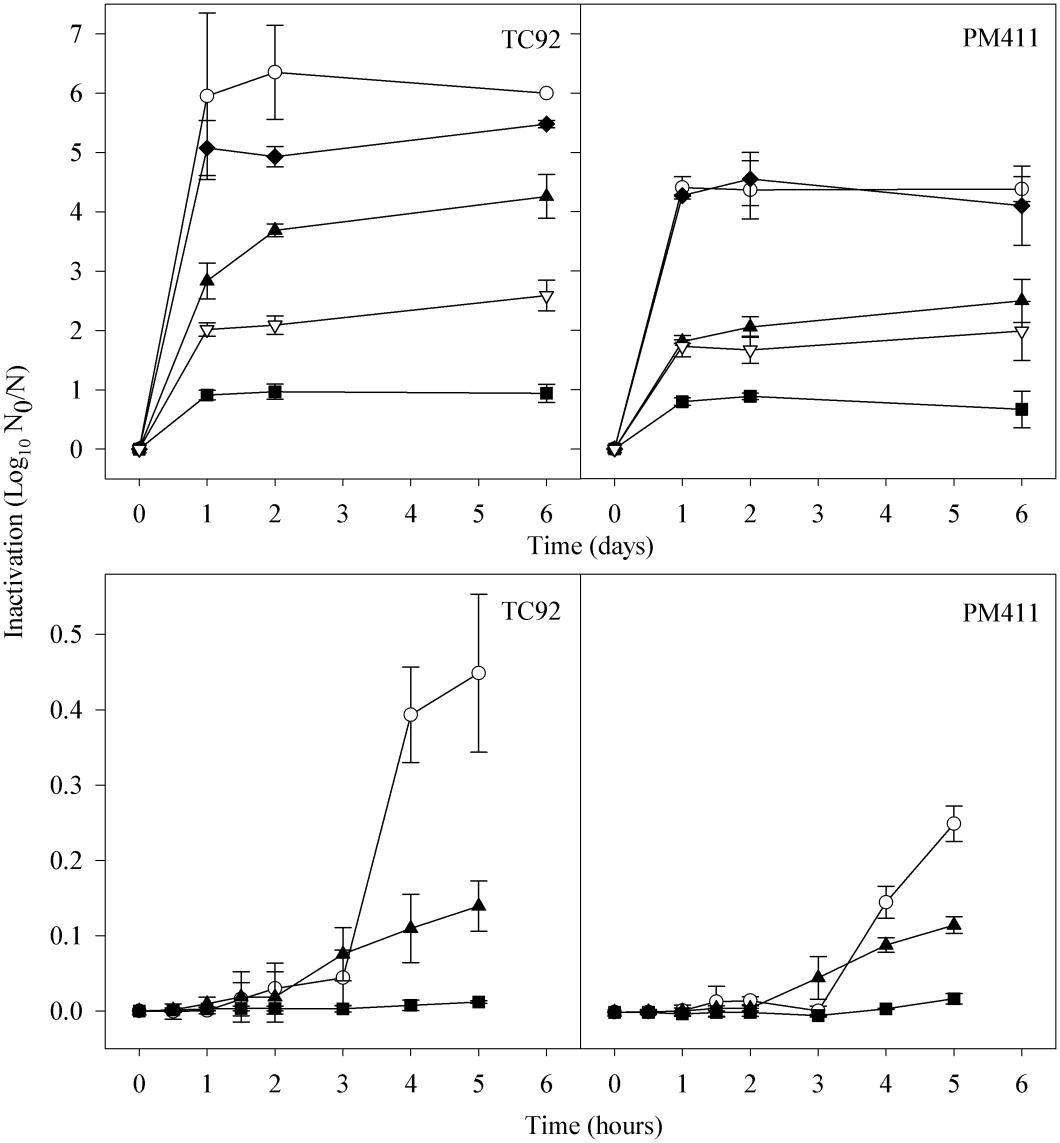
Effect of adaptation treatments on cell inactivation in *L*. *plantarum* TC92 and PM411 during desiccation. Long-term (top panels) and short-term (bottom panels) periods. Non-adapted, N (○), acid adapted, A (▲), hyperosmotic adapted, O (♦), stationary phase adapted, S (▽), and combined-stress adapted, C (■) cells. Inactivation is shown as log N_0_/N, where N_0_ is the initial number of cells and N is the number of survivors. Values are the mean of three independent biological replicates and error bars represent the standard deviation of the mean.

The behaviour of acidic (A), hyperosmotic (O), stationary phase (S), combined-stress (C) adapted and non-adapted (N) cells of PM411 and TC92 strains, during a long-term and a short-term period under desiccation stress was compared (Fig 2).

Over the long-term period of desiccation, the C adaptation treatment had the lowest inactivation values (less than 1 log after 6 days of desiccation), significantly different from the other adaptation treatments in both strains (*P* < 0.001). Inactivation values of A, S, and C cells were significantly lower than N cells in both strains (*P* < 0.001). S and A treated cells had inactivation values around 2 log in PM411, while in TC92 these values were around 4 log for A cells and 2 log for S cells. O treated cells had lower inactivation values than N cells in TC92 (specifically at 2 and 6 days under desiccation) but had similar inactivation in the case of PM411cells. Overall, PM411 N cells were less affected by desiccation stress than TC92 N cells.

The short-term experiment was performed once the best treatments were selected. Also in this case the inactivation values of A and C treated cells were significantly lower than in N cells in both strains (*P* < 0.001), being the C adaptation treatment which gave the lowest inactivation values. No significant differences (*P* > 0.05) were observed in inactivation among the treatments in both strains during the first 3 h. However, after this period, the inactivation values increased for A and N treatments. TC92 N cells reached higher inactivation values than PM411 N cells.

### Effect of adaptation treatments on survival on flowers and leaves under low relative humidity conditions

**Fig 3:**
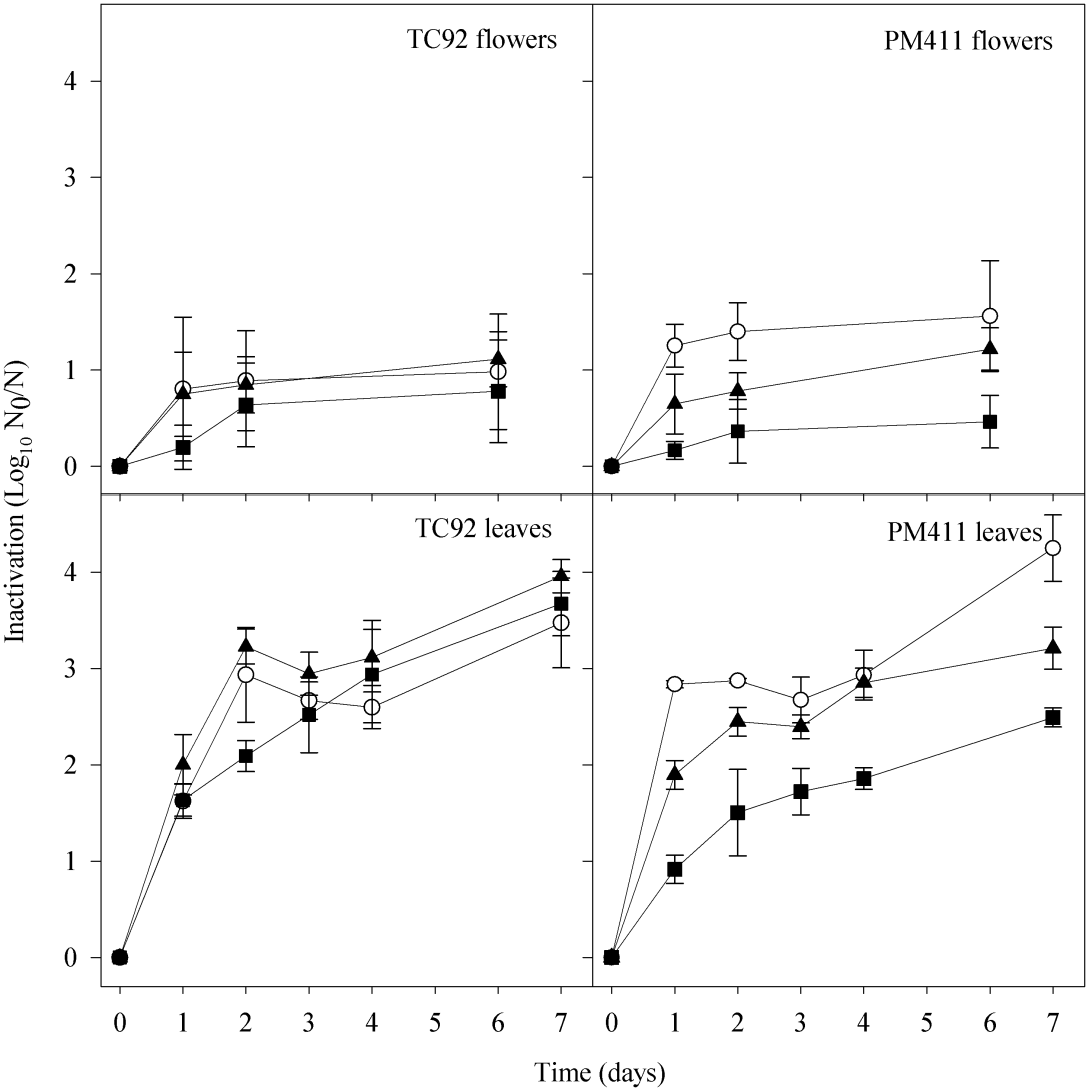
Effect of adaptation treatments on cell inactivation in *L*. *plantarum* TC92 and PM411 in apple flowers and *Prunus* leaves at low RH. Non-adapted, N (○), acid adapted, A (▲), and combined-stress adapted, C (■) cells. Inactivation is shown as log N_0_/N, where N_0_ is the initial number of cells and N is the number of survivors. Values are the mean of three independent biological replicates and error bars represent the standard deviation of the mean.

The inactivation of PM411 and TC92 previously submitted to adaptation treatments was studied in apple flowers and *Prunus* leaves at low RH (Fig 3).

In both strains, A, C, and N treated cells reached lower inactivation values on flowers (1.1 to 1.5 log at 6 days post inoculation) than on leaves (3.5 to 4.3 log at 7 days post-inoculation). In PM411, the inactivation of C treated cells was significantly lower than in A and N treated cells, in both plant materials (*P* < 0.001). Hence, the C treatment clearly improved PM411 cell survival, both on flowers and leaves. No significant differences in inactivation were observed between adapted and non-adapted TC92 cells, neither on flowers nor on leaves (*P* > 0.05) (except for C treated cells on leaves in day 2, which were significantly lower than A and N treated cells (*P* < 0.05)).

Globally, PM411 C cells showed lower inactivation than TC92 C cells. On leaf surfaces at low RH, inactivation values were 2 log in PM411 compared to 4 log in TC92. However, in blossoms, the inactivation in PM411 C adapted cells did not differ from TC92 cells.

### Transcriptional response to adaptation treatments and desiccation stress

**Fig 4:**
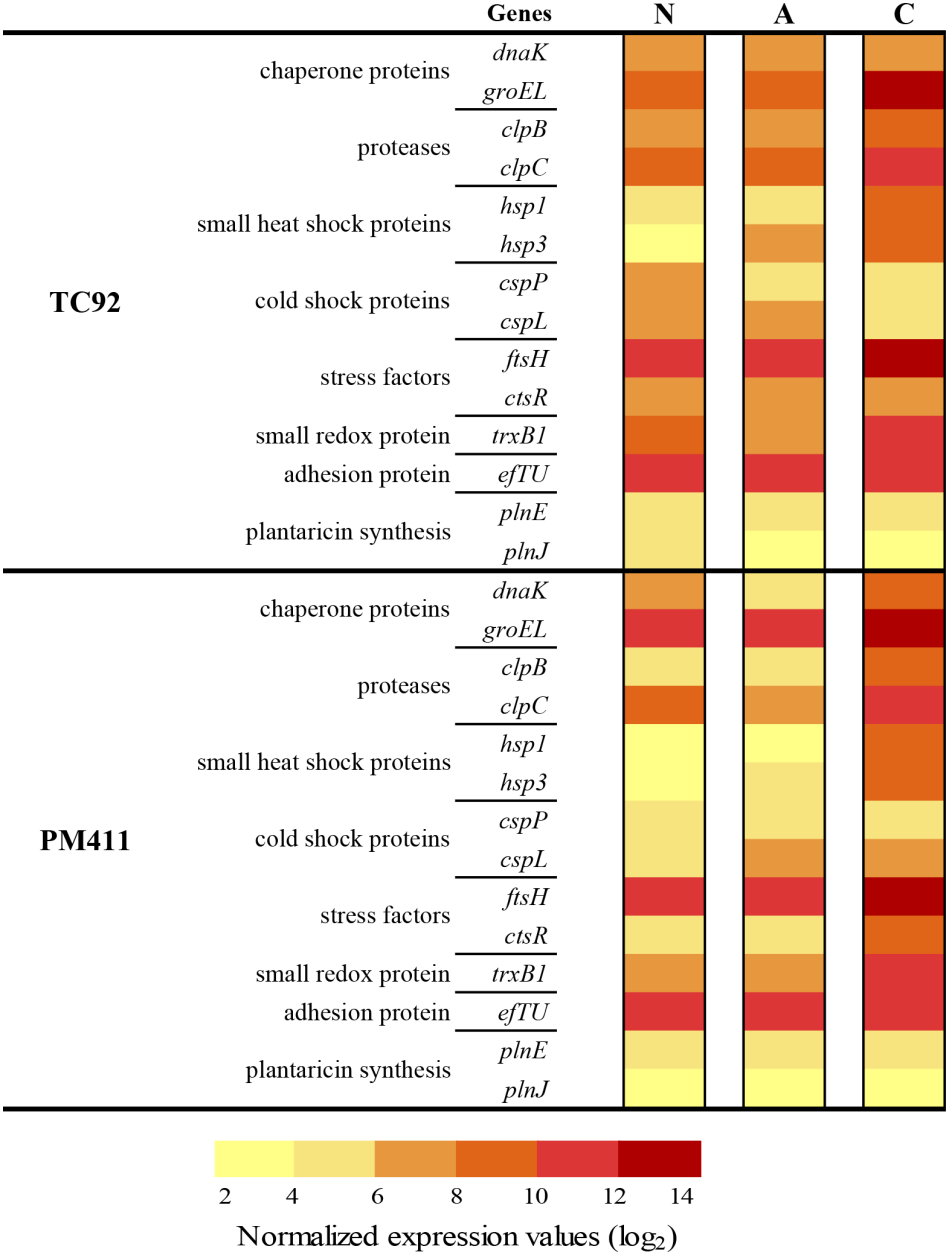
Transcriptional analysis during adaptation conditions in *L*. *plantarum* TC92 and PM411. Normalized expression values (log_2_) of genes associated to stress response, adhesion factor protein, and plantaricin synthesis, in non-adapted (N), acid adapted (A), and combined-stress adapted (C). *ldhD* gene was used for data normalization. Three independent biological replicates were performed.

The effect of adaptation treatments and desiccation stress on the expression levels and pattern of selected genes in PM411 and TC92 cells was studied. Gene expression during adaptation in acid (A), combined-stress (C), and non-adapted (N) conditions is shown in Fig 4. The expression pattern was quite similar in the two strains, but differed depending on treatments. A and N treatments showed similar expression pattern (in 10 out of 14 genes). However, the C treatment showed different expression levels in both strains (in 10 out of 14 genes) in comparison to A and N treatments. Expression values of stress related genes were higher in C than in N treated cells, for 10 genes in PM411 (*dnaK*, *groEL*, *clpB*, *clpC*, *hsp1*, *hsp3*, *cspL*, *ftsH*, *ctsR*, and *trxB1*), and for 7 genes in TC92 (*groEL*, *clpB*, *clpC*, *hsp1*, *hsp3*, *ftsH*, and *trxB1*). The adhesion factor *efTU* and the plantaricins *plnE* and *plnJ* had similar levels of expression in all treatments and in both strains. Interestingly, in C treated cells, in both strains, cold shock proteins (*cspP* and *cspL*) showed the lowest levels of expression of stress-related genes.

**Fig 5:**
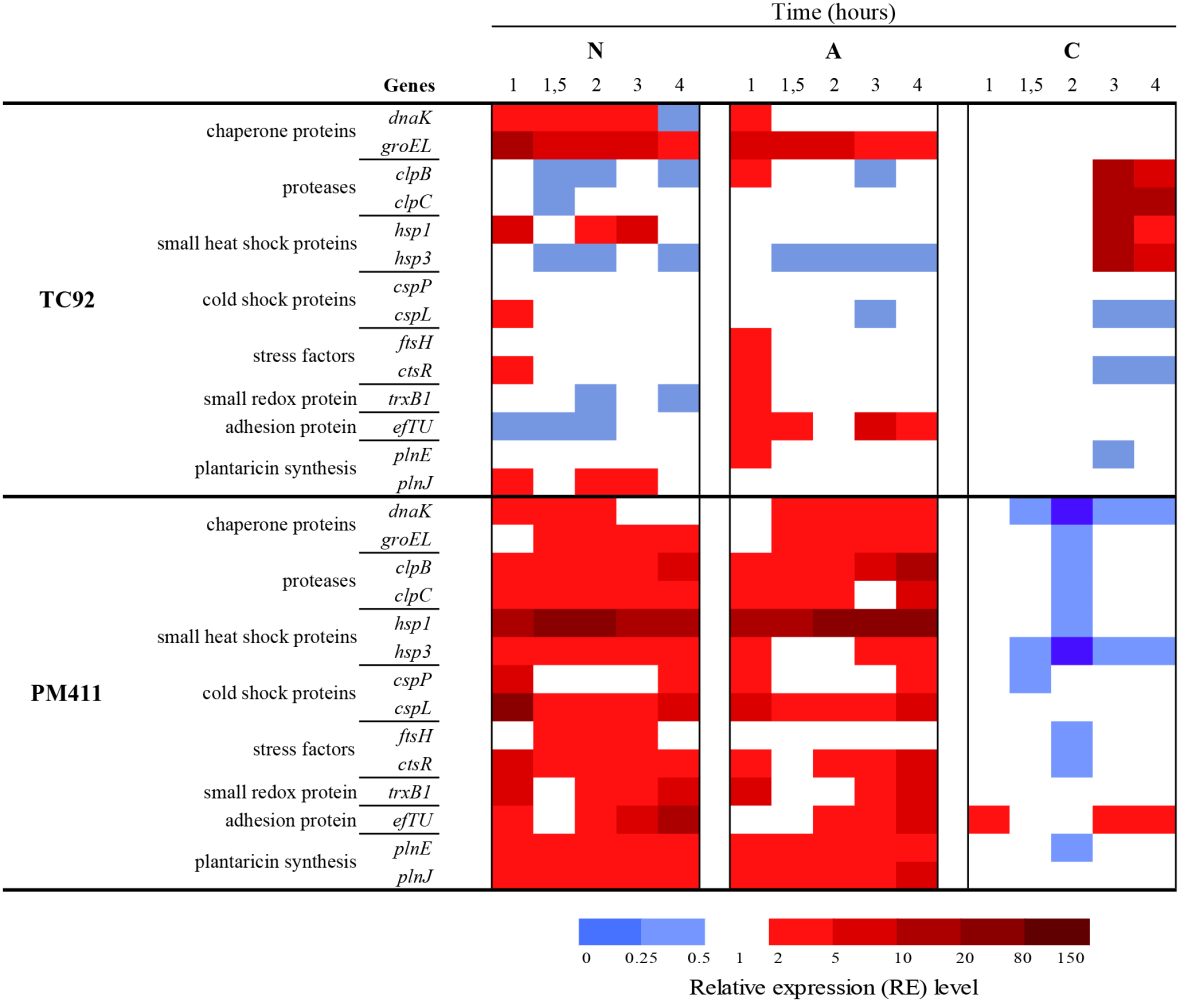
Transcriptional pattern of adapted cells of *L*. *plantarum* TC92 and PM411 during desiccation challenge. Non-adapted (N), acid adapted (A), and combined-stress adapted (C) cells. Samples during desiccation were taken at selected steps throughout a 4 h period. The relative expression (RE) level was assessed by the ΔΔCt method. The *ldhD* gene was used as the internal control. The ΔCt of non-desiccated cells for the corresponding adaptation method was defined as the calibrator. Three independent biological replicates were performed.

Gene expression during desiccation stress, depending on adaptation treatments, is shown in Fig 5. Non-adapted cells showed different desiccation stress response patterns in each strain. In PM411 N cells all genes were upregulated and, in general, their expression levels were kept upregulated over time. On the contrary, in TC92 N cells only *dnaK* and *groEL* genes were kept upregulated over time. So, TC92 N cells had a lower response capacity to desiccation stress than PM411. The transcriptional pattern of A treated cells did not differ significantly from N cells in both strains. Interestingly, the expression pattern of C treated cells was different from the N and A cells in both strains. Most of the genes were downregulated at some singular point or were kept unchanged during the desiccation stress period. However, the expression levels of some particular genes were actually different between TC92 C and PM411 C cells. For example, *clpB*, *clpC*, *hsp1* and *hsp3* were upregulated only in TC92 under desiccation conditions for 3 hours, and their expression level was kept upregulated during the following hour. The *efTU* gene was upregulated in PM411 C cells while was unaltered in TC92 C cells.

### Effect of adaptation on survival under greenhouse and field conditions and on efficacy of biological control

**Fig 6:**
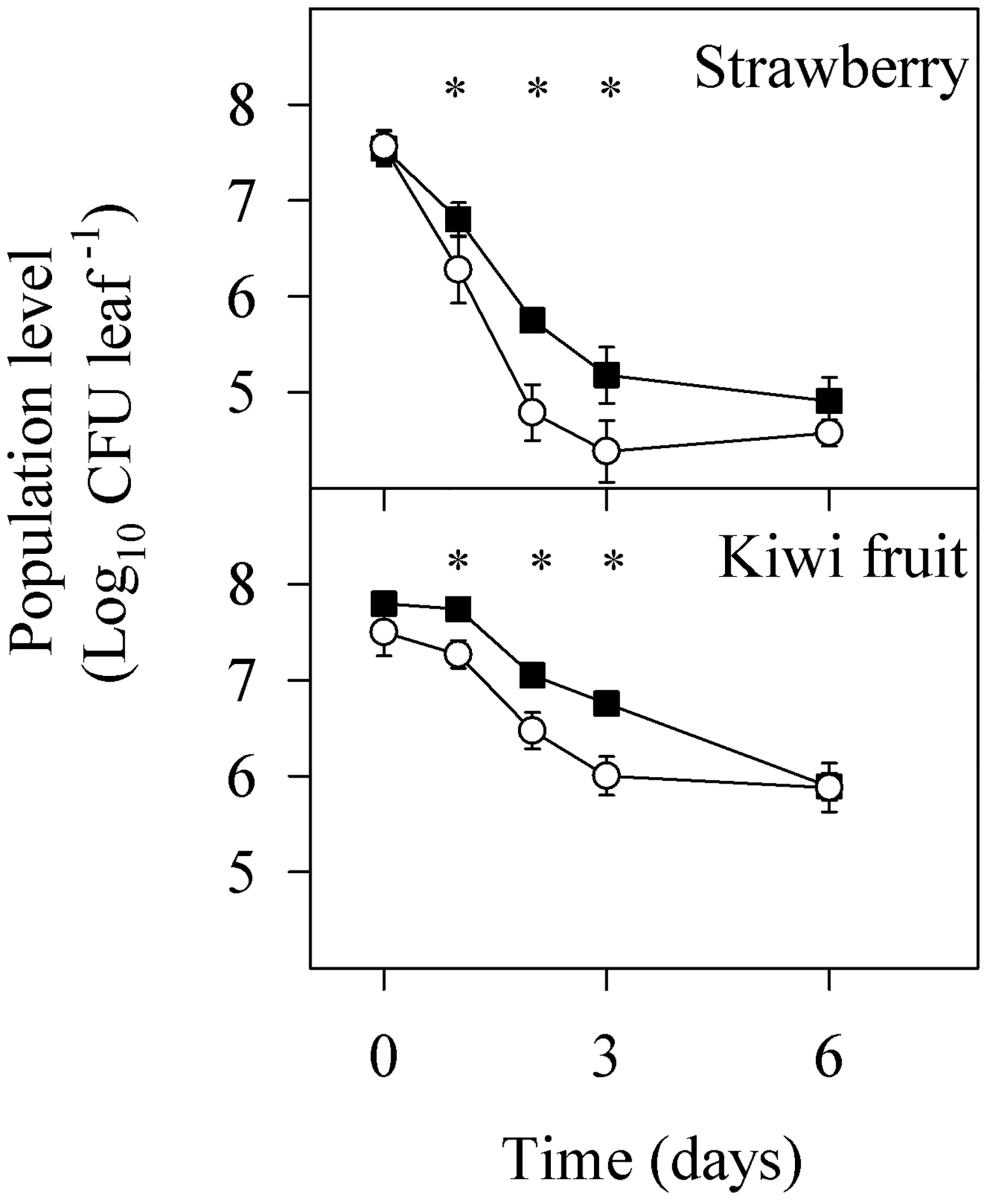
Effect of adaptation treatment on survival of PM411 in strawberry and kiwifruit leaves, under controlled environment greenhouse conditions. Plants were sprayed with non-adapted, N (○) or combined-stress adapted, C (■) cells. Values are the mean of three independent biological replicates and error bars represent the standard deviation of the mean. *, indicates significant differences between treatments according to the Tukey test.

Levels of PM411 on plants under greenhouse conditions decreased slightly through time and were higher in kiwifruit than in strawberry plants (Fig 6). Differences in population levels were observed between adapted and non-adapted PM411 cells in the two experiments performed for each plant species.

**Fig 7:**
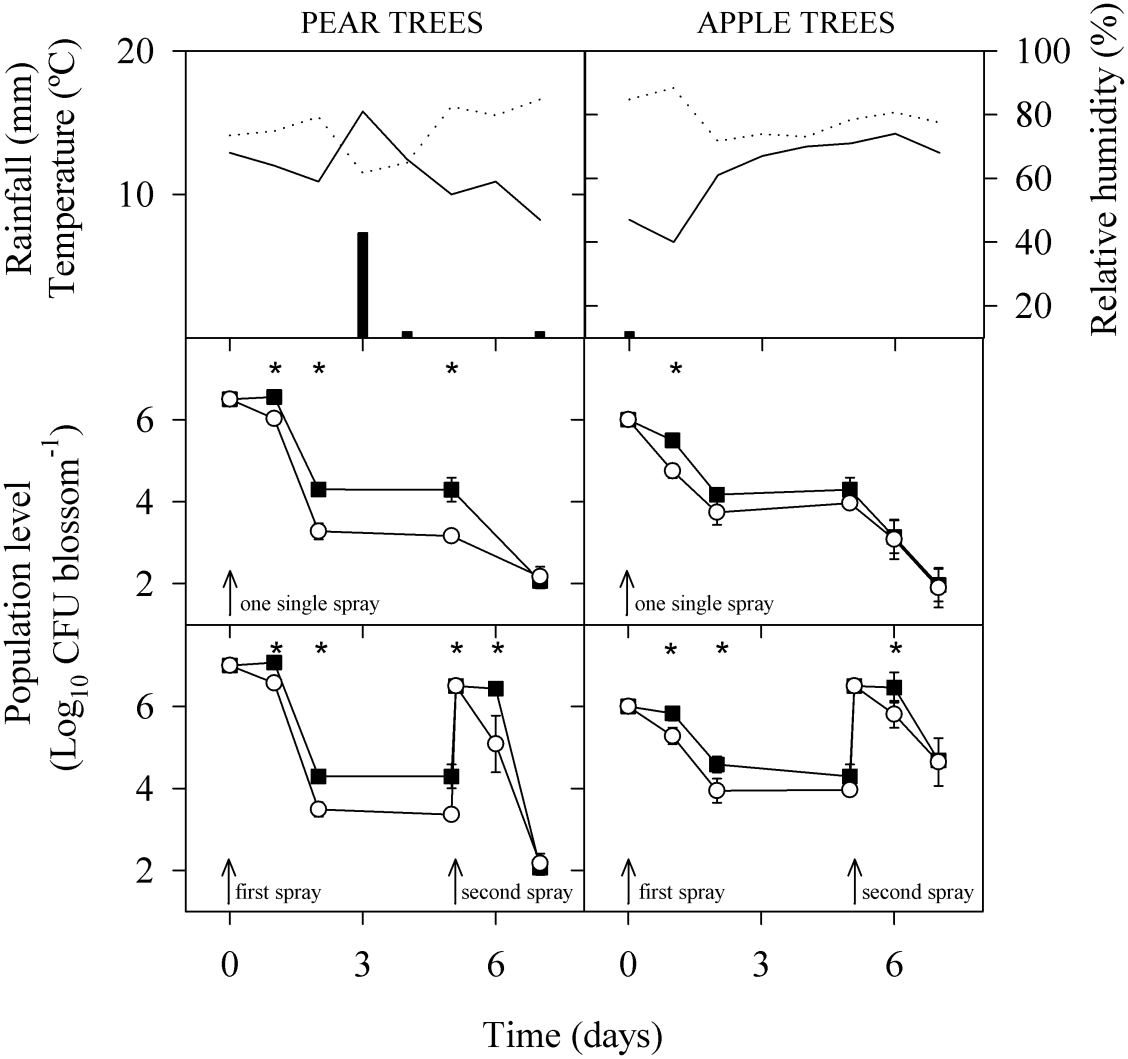
Effect of adaptation treatment on survival of PM411 in blossoms of ‘Comice’ pear and ‘Golden Smoothie’ apple under field conditions. Environmental conditions during the field experiments are shown in the upper panels. Temperature (dotted line), RH (bold line) and rainfall (vertical bars). Blossoms were treated in the field with either adapted cells (C) (■), or non-adapted cells (N) (○), using a single spray strategy or a repeated spray strategy. Values are the mean of three replicates. Error bars represent the 95% confidence interval of the mean. *, indicates significant differences between treatments according to the Tukey test.

Population levels of PM411 decreased also through time on pear and apple blossoms under field conditions that were relatively dry (one single rainfall event in pear tree assay and moderate temperatures and low humidity in both pear and apple trees; Fig 7). Three days following field inoculation, population levels of PM411 decreased to steady-state values in non-adapted treatments (10^3^–10^4^ CFU per blossom), but were significantly higher in adapted treatments (10^4^–10^5^ CFU per blossom) (Fig 7). When a second spray of PM411 was applied under harsh conditions, also adapted cells survived better than non-adapted cells on both, pear and apple plants.

**Fig 8:**
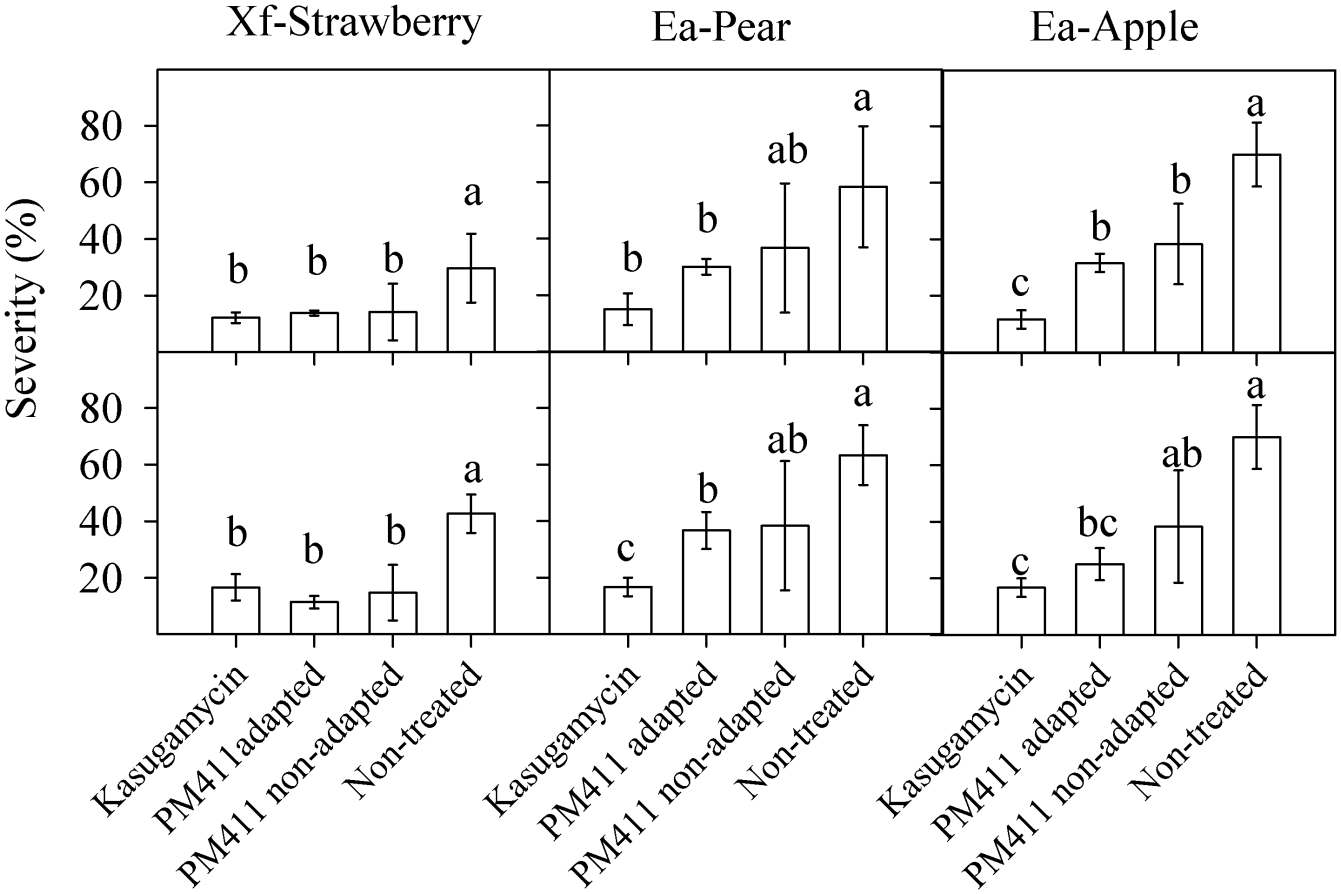
Effect of adaptation of PM411 on susceptibility of strawberry plants to infection by *X*. *fragariae* (Xf) and of pear and apple flowers to infection by *E*. *amylovora* (Ea). Plants and blossoms were treated under greenhouse and field conditions, respectively, with adapted PM411 cells, non-adapted PM411 cells, kasugamycin or non-treated. Strawberry plants were sprayed with Xf in the greenhouse. Once treated, pear and apple flowers were collected in the field and inoculated with *E*. *amylovora* under controlled environment conditions in the laboratory. Values of severity are the mean of three replicates. Error bars represent the 95% confidence interval of the mean. Bars for severity panels with the same letter in the same panel do not differ significantly (P<0.05) according to the Tukey test.

A decrease in disease severity was observed compared to the control in plants treated with both adapted (C) and non-adapted (N) cells of PM411, without significant differences between them (Fig 8). However, the C treatment had less variability compared to the N treatment and no significant differences between N and non-treated control could be detected in four out of six experiments performed. More in detail, the coefficients of variation in both experiments performed were for the C treatment 5.7 and 17.1% in strawberry, 8.3 and 15.7% in pear and 9.1 and 20% in apple, and those of the N treatment were 62.6 and 59% in strawberry, 55.1 and 52.7% in pear and 32.8 and 45.8% in apple. These results indicated that the C treatment gave more consistent efficacy.

## Discussion

In order to increase the tolerance to water stress of *L*. *plantarum* strains isolated from plant environments, and to improve its epiphytic survival under low relative humidity conditions, a strategy based on the adaptive growth under stress conditions has been developed. The combined-stress strategy consisted of adapting cells by growing them into a hyperosmotic medium until stationary-phase, which involves also acid production.

Our results with *L*. *plantarum* are in agreement with the findings in other *Lactobacillus* species from fermented foods, that reported a higher tolerance to stress of stationary-phase cells compared to exponential-phase cells [17, 19, 42, 56]. Also, the present results are in accordance with studies showing that cells obtained from non-controlled pH fermentations had better tolerance to stresses than cells from controlled pH experiments [57–59]. In addition, cells of *Lactobacillus* had enhanced tolerance to harsh conditions when grown under salt [60–62] or acid stress [20, 63].

The response of strains PM411 and TC92 toward the combined-stress treatment resulted in a strong protection against desiccation. The study of the gene expression responses immediately after the combined-stress treatment confirmed an increase in transcript levels of the stress-related genes (*DnaK*, *GroEL*, *ClpC*, *ClpB*, *Hsp1*, *Hsp3*, *CspP*, *CspL*, *CtsR*, *FtsH* and *TrxB1*), in comparison with non-adapted cells. Whereas, after desiccation challenge, transcript levels of the same genes remained unaltered or repressed in adapted cells while were overexpressed in non-adapted cells. This is in concordance with a better survival of adapted vs. non-adapted cells. These results suggest that cells after the combined-stress treatment have high levels of stress proteins that probably protect themselves from damage caused by a subsequent desiccation. Other studies performed in probiotic and dairy lactic acid bacteria have also shown that the adaptive response to stress involves the overproduction of several stress-related proteins, such as DnaK, GroEL, Clp, FtsH, that might give better survival under harsh environment [31, 43, 62, 64]. Interestingly, in our strains, the combined-stress adaptation treatment did not affect the expression level of the non-stress related genes *efTU*, *plnE* and *plnJ*. This is in agreement with the report that *plnE/F* and *efTU* gene expression in *L*. *plantarum* and *L*. *pentosus* was not affected by salt contents or pH variations in the growth medium [29, 32].

The acidic treatment, consisting of a single stress factor, showed less protection against desiccation than the combined treatment. In accordance, the gene expression pattern immediately after the acidic adaptation treatment and after desiccation challenge was quite similar to non-adapted cells in both strains. However, this can be a particular response of our strains, since other studies have shown differential expression of some genes in acid-adapted cells compared to non-adapted cells, specifically those related with malolactic fermentation and intracellular accumulation of histidine in *L*. *casei* [63] or stress-related genes (e.g. *clpL*) in *Oenococcus oeni* [22].

Differences in tolerance to desiccation were observed between PM411 and TC92 cells, in agreement with the gene expression patterns. PM411 non-adapted cells survived better than TC92 over the period of desiccation and showed upregulation of all the eleven stress-related genes analysed, whereas in TC92 only three genes were upregulated. Concerning the response to the combined adaptation treatment, both strains increased cell survival under desiccation but had slightly different stress-related gene expression patterns. After the adaptation treatment, PM411 showed overexpression in more genes than TC92. Moreover, throughout desiccation only in TC92 heat shock proteins (*hsp1* and *hsp3*) and proteases (*clpB* and *clpC*) were differentially upregulated after 3 h, probably due to cell damage and death, because it was at this time when inactivation in non-adapted cells increased.

In plant surfaces, under low RH conditions, the combined-stress adaptation treatment increased cell survival only in PM411. Therefore, we hypothesize that the differences in the stress-related gene expression patterns between the strains might be indicative of a better behaviour of PM411 adapted cells, with a high capacity to withstand stresses in plant surfaces. This is in agreement with the report on differences of tolerance to stress between strains of *L*. *plantarum* from fermented foods [56, 59]. In addition, differences in stress response between strains of *Pseudomonas fluorescens* antagonistic to *E*. *amylovora* have also been reported, which explained their performance in disease biocontrol [12, 55, 65]. Accordingly, in PM411 adapted with the combined treatment, the *efTU* gene was upregulated after desiccation challenge, while not changed in TC92. Interestingly, the *efTU* gene, which encodes an adhesion-like protein elongation factor, may play a role in adhesion. As reported, in *L*. *plantarum* the *efTU* gene is upregulated in the presence of mucus covering the intestinal surface suggesting its role in adhesion [29]. In addition, adhesion-like proteins were reported to be released from the cells of hiochi bacteria (*Lactobacillus* spp.) when they experience osmotic shock [28].

The combined-stress treatment improved survival of PM411 on plant surfaces under limiting environmental conditions (low RH) in different plant hosts. This beneficial effect was significant in *Prunus*, kiwifruit and strawberry leaves under greenhouse conditions, and in apple and pear flowers under controlled environment and also in the field. Under these unfavourable conditions, the strain did not multiply and only survived to steady-state populations, and adapted cells survive better than non-adapted cells. This effect was more important in leaves than in flowers. Probably this is because the water stress under low RH conditions is higher in leaves than in flowers. Leaves have waxy surfaces and are easily exposed to low RH conditions than flowers, and also provide limited nutrient resources to bacterial colonists. In contrast, flowers have internal structures (hypanthium and pistil) that can be colonized by *L*. *plantarum* where there are less prone to suffer sudden fluctuations in RH and have more nutrients available. Thus, bacteria may have more options of avoiding stresses in flowers than in leaves [14, 66]. The combined-stress treatment improved biocontrol efficacy since reduced variability within the trial, thus providing more consistency in disease suppression. A reduction of the within-experiment variability was also observed in other BCA and associated with an improvement of biocontrol [67]. This beneficial effect is related to the better survival performance of adapted PM411 on plant hosts.

Finally, strategies to adapt bacteria to stress conditions have been widely used in lactic acid bacteria to improve technological performance (fermented foods, probiotics), but not in the field of plant disease protection. Our results show that a combined adaptation treatment based on simultaneous hyperosmotic and acid stress, as well as cultivation until stationary phase, has beneficial effects on the survival of *L*. *plantarum* strains in response to desiccation and low relative humidity conditions on the plant surfaces. The effect was stronger in strain PM411 than in strain TC92, probably due to a better transcriptional response. This strategy increases PM411 survival on plant surfaces and the performance of biocontrol of bacterial diseases, thus, helping to develop improved microbial pesticides.
